# Can ChatGPT Reflect Professional HACCP Judgments? A Comparative Study in Hospitality Food Safety

**DOI:** 10.3390/foods15162916

**Published:** 2026-08-20

**Authors:** Despoina Maria Konstantinidi, Elisavet Stavropoulou, Agathangelos Stavropoulos, Chrysoula (Chrysa) Voidarou, Christina Tsigalou, Vassiliki Pitiriga, Christos Stefanis

**Affiliations:** 1 Laboratory of Hygiene and Environmental Protection, Medical School, Democritus University of Thrace, 68100 Alexandroupolis, Greece; 2 Department of Agriculture, School of Agriculture, University of Ioannina, 47100 Arta, Greece; 3 Department of Microbiology, Medical School, University of Athens, 11527 Athens, Greece

**Keywords:** AI, LLM, ChatGPT, HACCP, food safety, ISO, survey, hospitality sector, digitalization

## Abstract

The implementation of Hazard Analysis and Critical Control Points (HACCP) systems remains a cornerstone of food safety management. However, their effectiveness is influenced by organizational, human, and technological factors. This study investigates professional perceptions of HACCP implementation and explores the extent to which a large language model (LLM)—ChatGPT 4.1—can approximate human judgments in this domain. A structured questionnaire was administered to 90 professionals operating in food service, hospitality, industry, and consultancy. Responses were compared with outputs generated by ChatGPT 4.1 via a standardized multi-persona prompting protocol simulating five professional roles. To enhance response stability and minimize stochastic variation, each question was submitted in independent zero-shot sessions over multiple iterations. Responses were analysed across three thematic dimensions: barriers to HACCP implementation, perceived benefits, and digital readiness. Spearman correlation analysis of human responses revealed a systemic “training–turnover association,” where high staff turnover (r = 0.62, *p* < 0.01) was significantly associated with difficulties in maintaining continuous training. Statistical benchmarking using one-sample t-tests showed that the tested model generated significantly higher ratings for implementation barriers (*p* < 0.001) and digital readiness (*p* < 0.001) than the corresponding human assessments. These findings suggest that while ChatGPT can approximate aggregated professional perceptions in certain areas, notable divergences persist in operational and readiness-related domains. The study contributes methodological insights into human–AI comparative research and highlights opportunities and limitations of AI-supported decision-making in food safety management systems.

## 1. Introduction

Over the past decade, the regulatory landscape governing food safety in the European Union has evolved considerably, strengthening expectations for systematic hazard control and more rigorous verification of food safety practices across the hospitality and mass catering sectors. Between 2016 and 2025, successive updates to EU hygiene legislation and the modernisation of the farm-to-fork strategy shifted Hazard Analysis and Critical Control Points (HACCP) from a procedural compliance tool toward a performance-oriented framework that emphasises preventive control, documentation accuracy and continuous improvement. These changes have particularly affected operationally complex environments such as hotels and large catering units, where multiple production sites complicate the consistent application of HACCP procedures [1,2,3,4,5,6,7,8].

**Figure 1:**
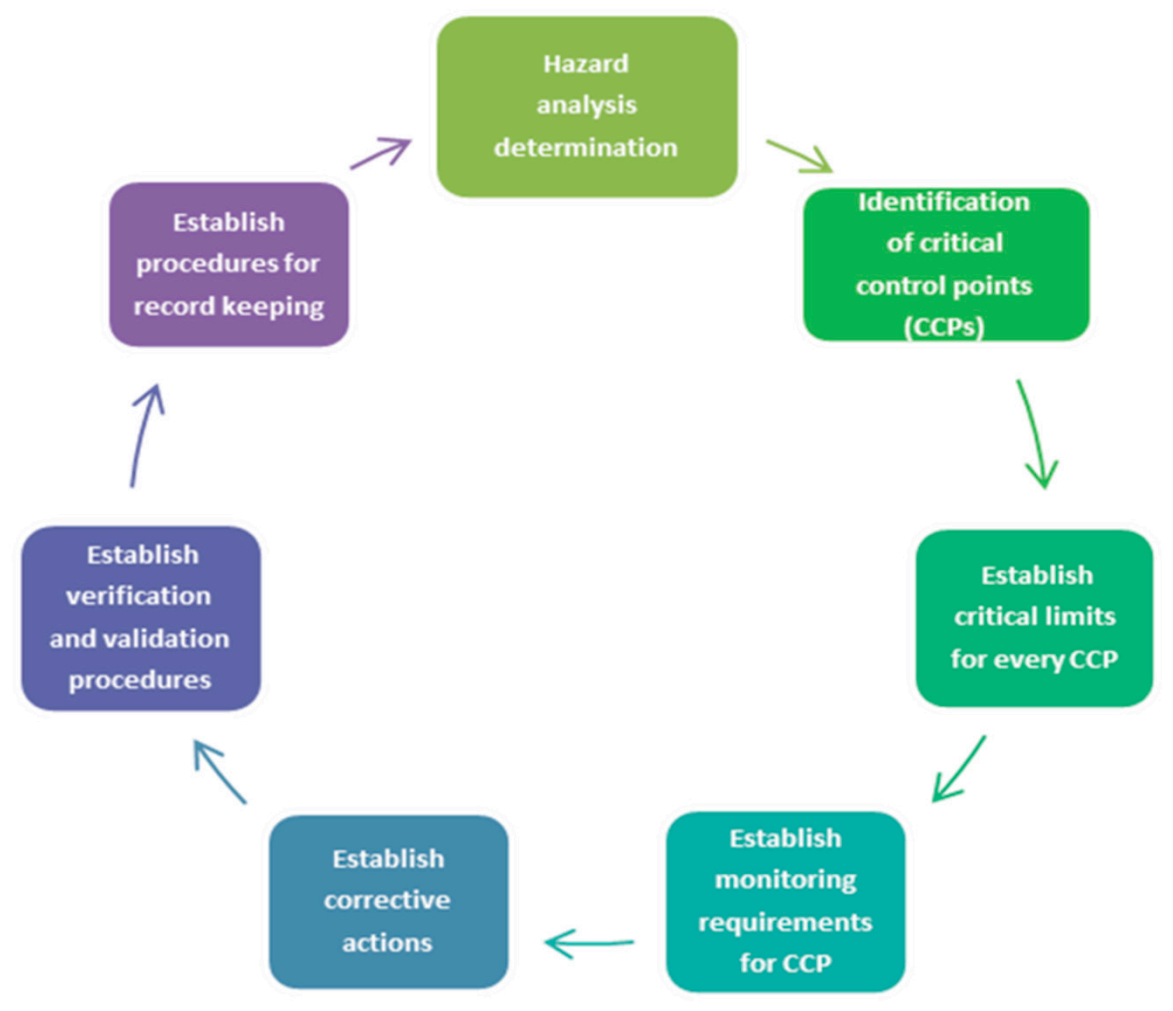
Flow diagram illustrating the sequential implementation of the seven HACCP principles in food safety management [15].

The HACCP (Figure 1) system constitutes the primary international framework for ensuring food safety in food service and hospitality environments. Numerous studies conducted in Europe and globally have demonstrated that the degree to which HACCP is successfully implemented depends heavily on staff training and competence, the overall food safety culture within an establishment, and the commitment of management to continuous enforcement of the system [9,10,11]. Despite its widespread regulatory adoption, substantial variability in compliance persists across food service and hotel operations, suggesting that legislative requirements do not necessarily translate into consistent day-to-day practice [12,13,14].

In parallel with regulatory developments, increased attention has been directed toward the financial and reputational consequences of foodborne disease outbreaks in hospitality settings. Evidence from recent years indicates that food safety failures may lead not only to direct economic losses but also to long-term reputational damage, intensified inspections, and operational disruptions, particularly in complex food service environments such as hotels and large catering units [16,17]. These risks have reinforced the importance of robust HACCP systems as core components of organizational risk management strategies within the hospitality sector.

Alongside these structural challenges, advances in digital technologies and computational risk assessment have introduced new opportunities for supporting food safety management. In particular, large language models (LLMs) and related artificial intelligence (AI) tools have demonstrated the capacity to process unstructured textual data, synthesize regulatory information, and identify potential food safety signals in diverse domains [18,19,20]. While such technologies have shown promise in decision support and analytical applications, the literature consistently emphasises that their outputs require careful interpretation, reproducibility controls, and alignment with domain-specific expertise [21,22].

Recent developments in large language models (LLMs) have shown their capacity to synthesize domain-specific information and produce structured technical reasoning. In food safety management, where professional decisions frequently rely on practical experience and contextual interpretation of guidelines, examining whether LLM-generated responses align with the perceptions of professionals involved in HACCP implementation is of increasing research interest. This perspective allows the evaluation of whether AI-generated reasoning reflects real operational conditions or primarily reproduces generalized regulatory logic.

Within this context, the present study provides a structured comparative assessment between the perceptions of professionals involved in food service and hospitality operations and the responses generated by a large language model. By examining areas of convergence and divergence between human judgments and LLM outputs across key HACCP dimensions, the study aims to contribute to the understanding of the perceptual alignment, limitations, and potential role of AI-based simulations as complementary tools in food safety management and professional training.

Accordingly, the study tested the following hypotheses: (H1) Professional perceptions of HACCP implementation barriers, benefits, and AI/digitalization differ across professional roles; (H2) LLM-generated assessments differ from human professional assessments across the examined HACCP dimensions; and (H3) Significant associations exist among selected organizational, implementation, and AI-related perceptions within the human dataset. These hypotheses were examined within the exploratory framework of the study, without implying causal relationships.

## 2. Materials and Methods

### 2.1. HACCP Questionnaire Design and Data Collection Procedures

The questionnaire was developed to systematically investigate perceptions, experiences, and practical aspects related to the implementation of the Hazard Analysis and Critical Control Points (HACCP) system in food service establishments and hotel operations (Supplementary Material S1). Its design was informed by contemporary methodological frameworks for KAP (Knowledge, Attitudes, Practices) studies in food safety and public health research, drawing on recent evidence from the food service and food technology literature [23,24,25,26].

The development process followed a multi-stage structure. An initial pool of items was generated based on international literature concerning HACCP implementation, regulatory compliance, operational barriers, and organizational food safety culture [27,28]. These items were organized into three sections:

- Barriers to HACCP implementation,
- Benefits of HACCP implementation,
- AI and Digitalization.

This thematic structure is consistent with contemporary multi-factor survey instruments used in food industry research [23,24].

The first validation stage involved a qualitative professional review by four specialists (n = 4) with expertise in food safety, food technology, hospitality management, and quality assurance. Reviewers assessed the clarity, relevance, conceptual structure, and coherence of the proposed items [23,24,29]. A formal quantitative Content Validity Index (CVI) was not calculated, as this stage was designed as a qualitative content review rather than a psychometric content-validity assessment. Based on the reviewers’ feedback, items requiring clarification were revised with respect to wording, terminology, and conceptual consistency. Prior to dissemination, a pilot test (n = 10) was subsequently conducted with food service and hospitality professionals to assess question comprehension, thematic flow, terminology precision, questionnaire functionality, and completion time [23,30].

The pilot sample was intentionally limited to 10 participants because its purpose was not statistical or psychometric validation but rather qualitative pre-testing of questionnaire comprehensibility, terminology, thematic flow, functionality, and completion time before full-scale dissemination. Methodological guidance indicates that approximately 10 participants, or even fewer, may be sufficient when pilot testing focuses on item wording, clarity, formatting, and ease of administration. Cognitive questionnaire pre-testing commonly employs small samples of approximately 5–15 participants per testing round. Feedback from the pilot resulted in further refinement of item wording, simplifications of technical terminology for non-specialists, and the inclusion of response options suitable for academic or research-oriented participants who evaluate HACCP conceptually rather than operationally [31,32].

The finalised questionnaire was distributed via Google Forms between 1 April and 30 April, allowing participants with professional or research experience in HACCP to complete the survey anonymously and voluntarily [23,24].

A purposive sampling strategy was employed, targeting individuals with professional or research experience related to HACCP—including food handlers, chefs and kitchen supervisors, quality managers, hotel F and B managers, HACCP consultants, and academic or research personnel. Distribution occurred through professional associations, hospitality networks, food service organizations, and academic channels.

The standardized format facilitated the reliable transformation of each item into a prompt and supported valid comparisons between human responses and model-generated outputs, in line with emerging methodological frameworks for human–LLM comparative research [21,22]. The operationalization of this process is described in detail in Section 2.2. The original Greek introductory text and the full questionnaire are provided in the Supplementary Material of this article.

### 2.2. Standardised ChatGPT Simulation Protocol and Comparative Statistical Analysis of Human and AI-Generated Responses

This study compares participants’ responses to the HACCP questionnaire in food service and hotel settings with those generated by the large language model ChatGPT 4.1. To capture stakeholder-specific perspectives, five distinct professional persona-based prompts were employed, simulating a multi-stakeholder expert panel (e.g., HACCP Consultant, Production Staff, Manager/Owner, Auditor, Researcher/Academic). Persona definitions were limited strictly to professional role framing and did not involve narrative role-playing, emotional context, or adaptive interaction. Each questionnaire item was administered in an independent, zero-shot session using standardized prompts to ensure consistency, neutrality, and comparability across personas. This standardized approach minimizes bias and ensures comparability between human and computational responses [22]. Zero-shot prompting was applied in the sense that no examples, prior answers, or iterative refinement were provided, and each response was generated independently. The prompts specified only the response format (e.g., a Likert 1–5 rating accompanied by a brief justification), following best practices outlined in recent methodological studies on human–AI comparative research [21]. Reproducibility was ensured by using identical prompts across all trials and submitting each question in an independent, memory-free session. For each professional persona, the prompt was executed in multiple independent runs (n = 30) to reduce stochastic variability and obtain a stable representative score. A fully standardized prompt structure and identical generation conditions were maintained across runs to minimize variability attributable to prompt formulation. The mean across repeated runs was therefore used as a stabilized computational reference for each questionnaire item. Importantly, this LLM-derived value was not interpreted as a population mean or population parameter, but as an experimental benchmark generated under the predefined simulation protocol. Accordingly, the one-sample t-test was used as an exploratory benchmark-based inferential procedure rather than as an estimation of an underlying LLM population parameter. The resulting *p*-values were therefore interpreted only as evidence of divergence between the distribution of human ratings and the predefined computational benchmark under the specified prompting conditions, while the direction and magnitude of divergence were assessed descriptively through Δ values.

Correlation analysis using a Spearman coefficient was applied exclusively to the human dataset to assess internal coherence and structural associations among questionnaire items, whereas alignment between human and ChatGPT responses was examined through descriptive comparisons and one-sample *t*-tests. Cronbach’s α was calculated exclusively for the human dataset to assess the internal consistency of the questionnaire scales [25,33]. The coefficient was not computed for the computational dataset, as ChatGPT 4.1 provides a single deterministic answer per question without within-item variance. Therefore, no multi-observation sample exists to permit a perceptual alignment estimate [21]. LLM mean scores represent the average of responses generated across the five professional personas for each questionnaire item. Because ChatGPT 4.1 outputs are probabilistic, a standardized prompt and identical generation conditions were used to reduce variability and the resulting persona responses were aggregated to obtain a stable benchmark value.

Differences between ChatGPT 4.1 and human mean scores (Δ) were calculated to facilitate comparative interpretation. A one-sample t-test was applied to examine whether human participant mean scores differed significantly from a fixed AI-derived reference value. The LLM mean score for each item was treated as a benchmark rather than a sampled population. This approach allowed assessments of whether human responses statistically confirmed or deviated from AI-based expectations [22,26].

All LLM experiments were conducted in May 2026 after completion of the online questionnaire distribution, using GPT-4.1 through the ChatGPT web interface (OpenAI, San Francisco, CA, USA). No API was used, therefore API version, API-specific model snapshot/ID, or API settings are not applicable. The model designation displayed to the researchers through the interface at the time of inference was GPT-4.1. The complete prompts used for all five professional personas, together with the response format instructions, are provided in the Supplementary Material. Because the experiments were conducted through the ChatGPT 4.1 web interface, backend system instructions and API-level configuration parameters were neither researcher-controlled nor directly accessible and therefore cannot be reported as experimental settings.

**Figure 2:**
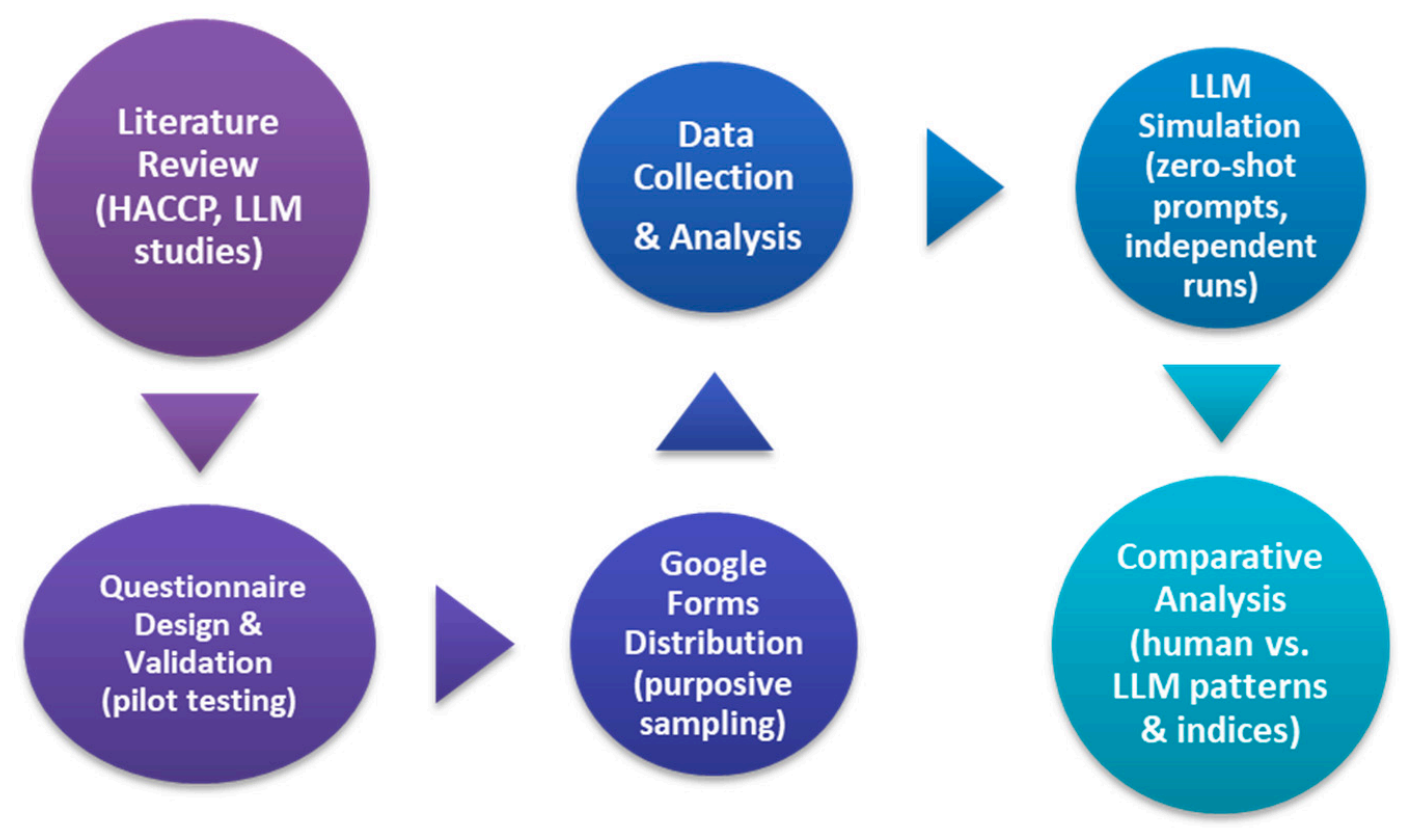
Flowchart of the study design, questionnaire development, LLM simulation protocol, and comparative analysis workflow.

Figure 2 presents the sequential stages of the study: literature review; development, professional validation, and pilot testing of the HACCP questionnaire; online data collection through purposive sampling; data cleaning and coding; statistical analysis of human responses; zero-shot prompt–based LLM simulation using ChatGPT 4.1; and the final comparative evaluation of human and AI-generated responses.

## 3. Results

### 3.1. Professional Survey Outcomes and ChatGPT Simulation Outputs

The results of the ChatGPT 4.1 Simulation Protocol indicate that the model consistently produces high scores across all sections of the questionnaire, reflecting consistently high model-generated ratings across the examined items related to HACCP compliance, operational barriers, and the potential benefits of both HACCP and digitalization. As shown in Table 1, all five sets of simulated responses—corresponding to the selected professional profiles (“Consultant,” “Production Staff,” “Manager/Owner,” “Auditor,” and “Researcher/Academic”)—converge toward similarly high ratings regarding perceived barriers, perceived benefits, and the potential of artificial intelligence to support HACCP systems.

In Section B (Barriers), all profiles assign particularly high scores to human-resource-related challenges, such as lack of staff motivation (Q6) and high employee turnover (Q7), with means approaching or exceeding 4.5. The “Consultant” and “Auditor” profiles produced the highest scores for staff turnover, while “Auditor” and “Academic/Researcher” identified deficiencies in prerequisite programs (Q11), reflecting a more systemic and standards-oriented interpretation of HACCP obstacles. By contrast, the “Production Staff” profile assigns the highest value to the documentation burden (Q9), while the “Manager/Owner” profile consistently rates this specific barrier lower than the other groups (3.50).

Section C (Benefits) shows near-uniform and very strong agreement that HACCP improves legal compliance (Q14), reduces the likelihood of customer complaints (Q15), enhances business reputation and credibility (Q16), and fosters a food safety culture among employees (Q18). Although a small number of profiles exhibit scores slightly below 4.5—primarily the Production Staff profile—the overall distribution in Section C remains very high (4.30–4.97), indicating a strong, cross-role consensus on the perceived benefits of HACCP. Τhe “Researcher/Academic” profile showsthe highest agreement regarding the role of HACCP in promoting a food safety culture (4.93).

In Section D (AI/Digitalization), the five simulated professional profiles present consistently positive evaluations of the potential contribution of artificial intelligence to HACCP processes. Mean scores across questions Q19–Q22 range from 3.70 to 4.83, indicating substantial agreement on the usefulness of AI-driven tools in food safety management. The strongest endorsement appears for automated monitoring of CCPs and PRPs (Q20), which achieves the highest overall mean (4.65), with Auditors (4.83) and Researchers/Academics (4.80) expressing the greatest confidence. For AI-supported decision-making through Large Language Models (Q21), the highest values are again observed for the Researcher/Academic (4.73) and Auditor (4.67) profiles, suggesting a technologically receptive stance among roles traditionally engaged in analytical evaluation and regulatory oversight.

A similar pattern emerges for AI-assisted HACCP training (Q19), where Consultants (4.40) and Researchers/Academics (4.77) provide the most favourable assessments while Production Staff report the lowest value (3.97), indicating a more cautious orientation among personnel working primarily in operational settings. The same gradient is observed in willingness to participate in AI-based HACCP training programs (Q22), with Researchers/Academics showing the highest level of openness (4.60), followed by Auditors (4.33) and Managers/Owners (4.07), and again the lowest score assigned by Production Staff (3.70). Taken together, these findings demonstrate that enthusiasm for digital transformation increases with greater exposure to structured decision-making and decreases among roles whose daily responsibilities are dominated by manual tasks. The ChatGPT 4.1 simulation produced consistently high scores across all five professional profiles (overall mean X¯ = 4.50), indicating broad alignment with the principles and perceived value of HACCP.

In Section B, human-resource-related barriers—particularly high staff turnover (Q7, X¯ = 4.77) and limitations in continuous training—were rated as the most critical across profiles. The second largest discrepancy concerned the documentation burden (Q9), to which Production Staff assigned the highest importance (4.83) and Managers/Owners the lowest (3.50), reflecting contrasting operational versus strategic viewpoints. Section C showed near-unanimous agreement, with all benefit-related items exceeding a mean of 4.50. The strongest overall benefit concerned enhanced business reputation (Q16, X¯ = 4.80), while the Researcher/Academic profile placed the highest emphasis on promoting a food safety culture (Q18, 4.93). In Section D, attitudes toward digitalization diverged: Auditors and Researchers/Academics demonstrated the highest confidence in AI-supported monitoring and decision-making, whereas Production Staff expressed the lowest willingness to engage in AI-based training (Q22, 3.70).

The study sample consisted of 90 professionals (n = 90) active in the food safety and hospitality sectors in Greece. The demographic and professional characteristics of the participants are summarized in Table 2.

A total of 90 respondents participated in the study, representing a diverse range of professional roles and levels of experience related to HACCP implementation. With respect to experience in HACCP, the majority of participants reported substantial professional exposure: 37% had more than 10 years of experience, while 22% reported 4–7 years and 18% reported 8–10 years of experience. Participants with limited experience were fewer, with 15% reporting 1–3 years and 8% reporting less than 1 year of involvement in HACCP-related activities.

Regarding organization type, respondents were primarily employed in mass catering or catering units (42%) and hotel units (35%), reflecting the study’s focus on hospitality and food service settings. Smaller proportions were affiliated with consulting or auditing firms (12%) and public authorities or regulatory bodies (11%), providing additional regulatory and advisory perspectives. In terms of training background, a large majority of participants (82%) reported having received certified HACCP training, while 18% indicated no official certification, suggesting that most responses were informed by formal training and professional qualification.

The largest groups consisted of HACCP consultants or auditors (n = 33) and quality or food safety managers (n = 32). Additional roles included managers or business owners (n = 10), researchers or academics (n = 7), and production or kitchen staff (n = 5), while a small number of respondents (n = 3) reported other food safety–related positions, such as regulatory authority staff or inspectors.

Finally, participants reported professional activity across multiple geographical regions of Greece, with the highest representation from Attica (n = 38) and Central Macedonia (n = 24). Smaller numbers were recorded from Eastern Macedonia and Thrace (n = 6), the Peloponnese (n = 5), Crete (n = 4), and the Ionian Islands (n = 4), while nine respondents reported activity in other regions, including Central Greece Regions, the North and South Aegean, Thessaly, Western Greece, Epirus, and Western Macedonia.

The results derived from the human questionnaire responses indicate a high level of overall agreement regarding both the challenges and benefits associated with HACCP implementation, while also revealing meaningful variations across professional roles, particularly in relation to operational constraints and digital transformation (Table 3).

In Section B (Barriers), the highest mean scores consistently relate to human resource–driven challenges. High staff turnover (Q7) emerges as the most critical barrier overall (Mean = 4.59), with particularly elevated scores reported by Auditors (4.70) and Researchers/Academics (4.65). Similarly, the inability to ensure continuous training (Q12) is rated as a major obstacle (Mean = 4.42), underscoring the dependence of HACCP systems on sustained knowledge transfer and skill development.

Issues related to staff motivation and commitment (Q6) and management support (Q10) also receive high evaluations, with mean scores exceeding 4.3. Auditors and Researchers assign greater importance to management commitment (4.55 and 4.45, respectively), reflecting a more systemic understanding of HACCP performance as an outcome of organizational governance rather than isolated operational practices. In contrast, the lack of specialized technical consultants (Q13) records the lowest overall mean (3.63), indicating that while external expertise is valued, it is perceived as less critical than internal organizational and human capital factors.

In Section C (Benefits), near-unanimous agreement is observed across all professional profiles. All items yield mean scores above 4.5, confirming a strong shared perception of HACCP as an effective and value-adding system. The highest overall rating is observed for improved compliance with legislation (Q14) (Mean = 4.82), with Auditors and Researchers/Academics assigning almost maximal scores (4.90 and 4.95, respectively). Benefits related to business reputation and credibility (Q16) and customer trust (Q17) are also rated extremely highly across all roles, highlighting that HACCP is widely perceived not merely as a regulatory obligation but as a strategic asset. The promotion of a food safety culture (Q18) shows slightly greater variation, with Researchers/Academics assigning the highest value (4.80), consistent with a longer-term and more conceptual perspective on organizational change.

Section D (AI and Digitalization) reveals a more nuanced pattern. While overall perceptions remain positive, mean scores are comparatively lower than those observed for HACCP benefits. The potential of AI to support HACCP training (Q19) and automated monitoring of CCPs and PRPs (Q20) receives favourable evaluations (Means = 4.10 and 4.31, respectively), particularly from Auditors and Researchers/Academics. In contrast, Production Staff consistently report lower scores, most notably regarding LLM-based decision support (Q21) (3.50), indicating greater scepticism toward AI systems that directly influence daily operational decision-making. Nevertheless, willingness to participate in AI-enabled HACCP training programs (Q22) remains relatively high across all groups (Mean = 4.19), suggesting openness to gradual digital integration when framed as supportive rather than substitutive.

### 3.2. Benchmarking Model-Human Consistency

**Figure 3:**
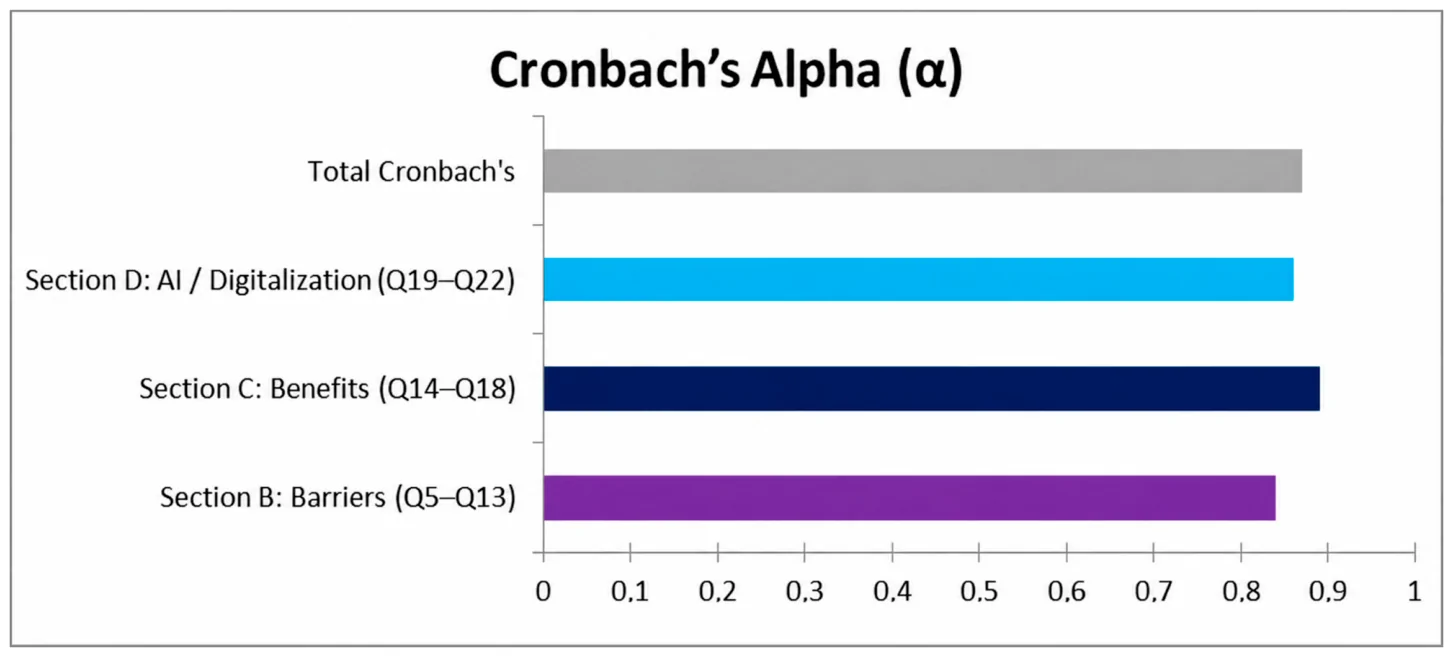
Internal consistency reliability of the questionnaire.

The below bar chart (Figure 3) displays Cronbach’s alpha (α) coefficients for the three main sections; Barriers to HACCP (α = 0.84), Perceived Benefits (α = 0.89), and AI/Digitalization Perspectives (α = 0.86) and the overall (Total) questionnaire. All values exceed the 0.80 threshold, indicating high perceptual alignment and professional-grade internal consistency for the study sample (n = 90).

**Figure 4:**
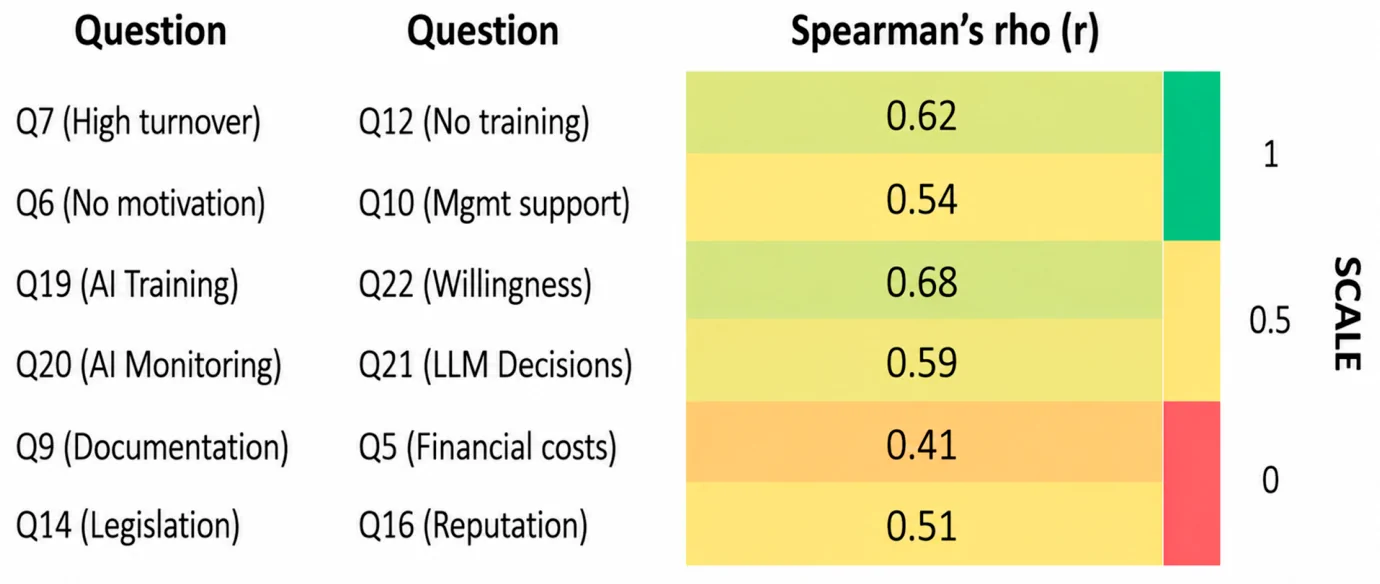
Statistically significant correlations between selected items of the human participant HACCP questionnaire (n = 90, *p* < 0.05).

Figure 4 presents statistically significant correlation coefficients (r) derived exclusively from the human participant HACCP questionnaire (n = 90). Colour intensity represents the strength of positive associations, ranging from weaker (red) to stronger (green) correlations. Only correlations reaching statistical significance at the *p* < 0.05 level are displayed.

The correlation analysis of the human participant HACCP questionnaire (n = 90) identified several statistically significant positive associations between selected survey items (*p* < 0.05). The strongest correlation was observed between AI training (Q19) and willingness to participate in AI-based training programs (Q22) (r = 0.68). A high correlation was also recorded between high staff turnover (Q7) and inability to ensure continuous training (Q12) (r = 0.62).

Moderate positive correlations were identified between lack of staff motivation (Q6) and management support and commitment (Q10) (r = 0.54), as well as between AI-based monitoring of CCPs and PRPs (Q20) and the use of Large Language Models for HACCP decision-making (Q21) (r = 0.59). Additionally, a moderate association was observed between improved legislative compliance (Q14) and enhanced business reputation and credibility (Q16) (r = 0.51). Finally, a statistically significant but weaker correlation was found between documentation burden (Q9) and financial constraints/equipment renewal costs (Q5) (r = 0.41).

Table 4 summarizes the mean scores of the human participants (n = 90) and ChatGPT-generated responses across all HACCP questionnaire items (Q5–Q22), along with the calculated difference Δ (LLM − Human). Overall, the tested model produced consistently high ratings across all dimensions, with an overall mean score of 4.51 compared to 4.31 for human respondents, indicating a general upward bias in model-generated evaluations.

In the Barriers to HACCP section, the LLM consistently produced higher mean scores compared to human respondents across almost all items, indicating a systematic tendency to assign greater weight to structural and organizational constraints. The largest positive deviations were observed for financial constraints and equipment renewal costs (Q5), lack of specialized technical consultants (Q13), and insufficient prerequisite programs (Q11), where the ChatGPT exceeded human ratings by more than 0.45 points. Moderate positive differences were also recorded for limited technical expertise of staff (Q8) and inability to ensure continuous training (Q12). In contrast, time required for documentation and record keeping (Q9) was the only barrier item for which the LLM assigned a lower score than human participants, suggesting a divergence in the perceived relative importance of administrative burden. Overall, the results indicate that while both humans and the ChatGPT 4.1 identify similar barriers, the model tends to amplify the perceived severity of resource- and infrastructure-related challenges.

In the Benefits of HACCP section, human and LLM assessments demonstrated a high degree of convergence, with all absolute differences remaining small. Human respondents marginally rated legislative compliance (Q14), reduction of customer complaints (Q15), and customer satisfaction and trust (Q17) higher than the ChatGPT 4.1, whereas the model slightly exceeded human scores only for the promotion of food safety culture among employees (Q18). The remaining item, enhancement of business reputation and credibility (Q16), showed near-identical ratings between the two sources. These results indicate strong alignment between human expertise and LLM-generated evaluations regarding the perceived benefits of HACCP implementation, with no item exhibiting a substantial divergence.

In the AI and Digitalization section, the tested model consistently assigned higher scores than human respondents across all AI-related functional items, reflecting a clear directional difference in assessment. The most pronounced discrepancy was observed for the role of ChatGPT 4.1 in supporting HACCP decision-making (Q21), followed by AI-based automatic monitoring of CCPs and PRPs (Q20) and the contribution of AI to staff HACCP training (Q19). These differences suggest that the LLM attributes greater perceived effectiveness to advanced digital and AI-driven applications than human professionals. Notably, willingness to participate in AI-based HACCP training programs (Q22) showed complete agreement between human and LLM scores, indicating convergence at the attitudinal level despite divergence in functional assessments.

**Figure 5:**
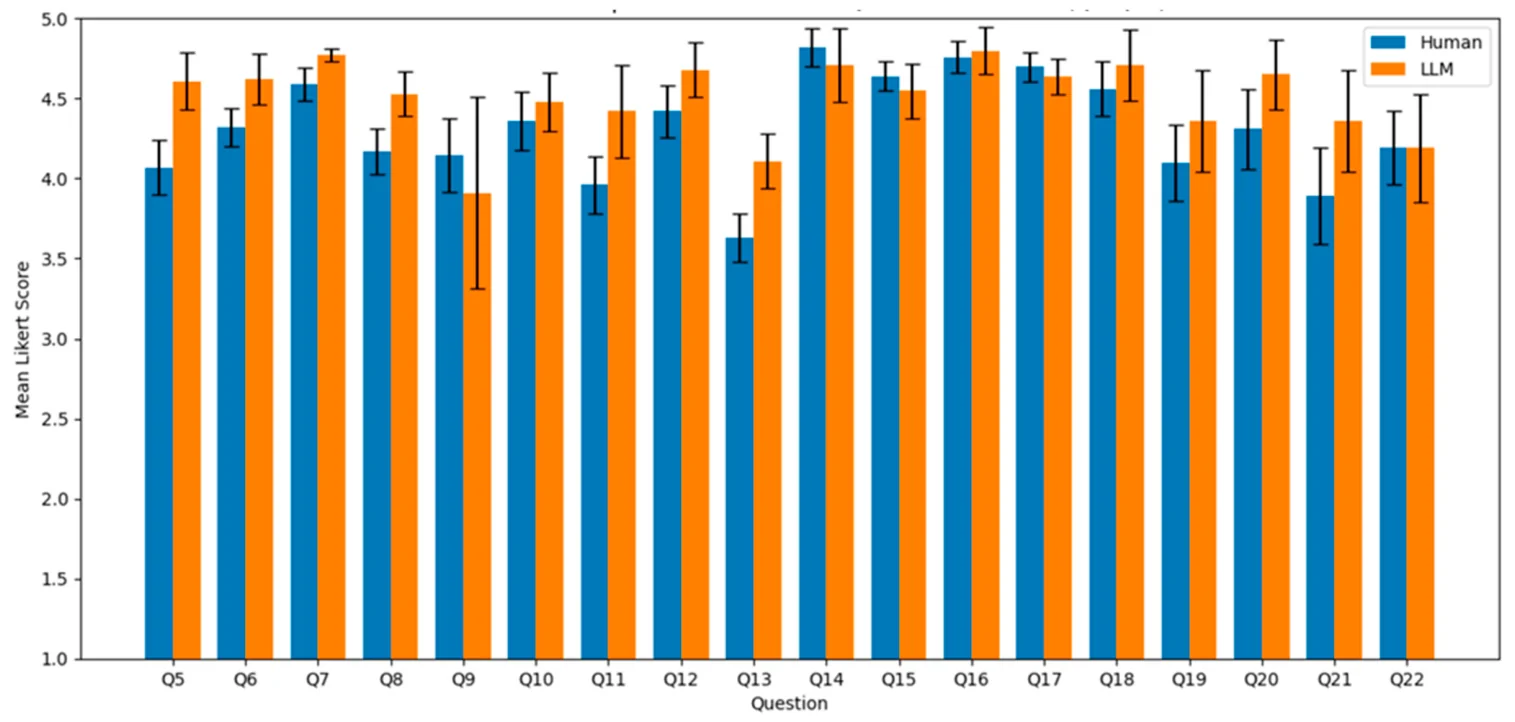
Comparison of human and LLM (ChatGPT 4.1) responses across HACCP questionnaire items (Q5–Q22). Bars represent mean Likert scores for human participants and LLM-generated responses. Error bars illustrate variability across professional roles.

Figure 5 presents a graphical comparison between human and LLM (ChatGPT 4.1) mean scores, including variability across professional roles, allowing visual assessment of agreement patterns and response dispersion.

**Figure 6:**
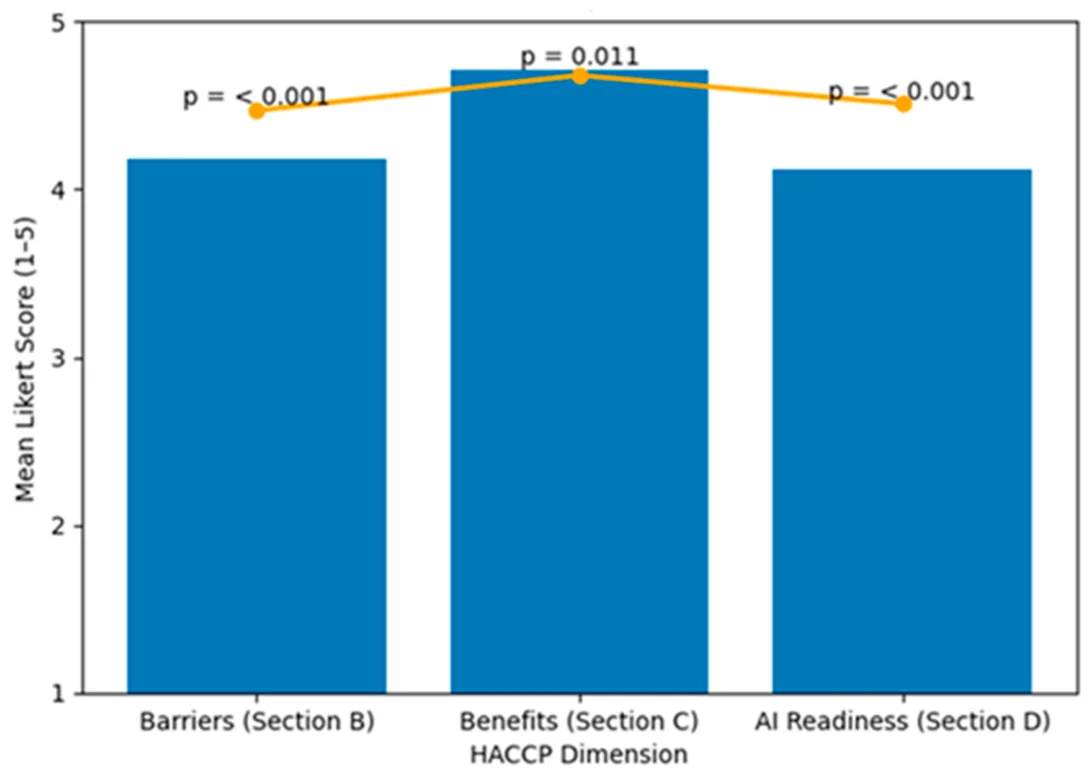
One-sample t-test results comparing human questionnaire responses (n = 90) against AI-derived benchmark values across HACCP dimensions (Mean Likert score, 1–5; *p* < 0.05).

Figure 6 represents mean Likert scores (1–5) reported by human participants for each HACCP dimension, while points indicate the corresponding AI-derived benchmark values used as test references in the one-sample t-tests. The ChatGPT 4.1 output was treated as a fixed reference value rather than a sampled distribution; thus, variance was derived solely from human responses. *p*-values denote statistically significant deviations between human responses and the AI benchmark.

Figure 6 summarizes the results of the one-sample *t*-tests comparing human questionnaire responses with AI-derived benchmark values across the three HACCP dimensions. Statistically significant deviations were observed for the Barriers to HACCP (Section B) and AI Readiness (Section D) dimensions, where human mean scores were significantly lower than the LLM reference values (*p* < 0.001 for both sections). In contrast, for the Benefits of HACCP dimension (Section C), human responses were slightly but significantly higher than the AI benchmark (*p* = 0.011), although the absolute difference in mean scores was minimal.

## 4. Discussion

Several studies conducted in Southern Europe and Greece have consistently reported persistent gaps between formal HACCP adoption and its effective operational implementation [34,35,36,37]. The findings of the present study reinforce this body of evidence, as the elevated scores observed in the Barriers section (Average: 4.18) are consistent with previously documented infrastructural deficiencies, limited technical expertise, and organizational constraints in small and medium-sized hospitality establishments [38,39,40]. In hotel environments in particular, the structural complexity of food production systems, combined with seasonal workload fluctuations, further challenges the stability of HACCP monitoring and verification procedures [41,42,43,44,45]. Within the present Greek sample, ChatGPT 4.1-derived ratings were higher than the corresponding human ratings for several HACCP implementation barriers.

A relatively strong positive association was observed between high staff turnover and difficulty in ensuring continuous training (r = 0.62), indicating that these barriers tend to co-occur. Given the cross-sectional design, however, the direction of this relationship cannot be established. Therefore, the finding should not be interpreted as evidence that turnover disrupts training or that insufficient training increases turnover. Correlation analysis of human responses revealed a strong association between high staff turnover and the inability to ensure continuous training (r = 0.62, *p* < 0.01), empirically substantiating earlier qualitative observations that workforce instability undermines long-term food safety capacity building [41,46]. In parallel, the significant relationship observed between lack of staff motivation and insufficient management support (r = 0.54, *p* < 0.01) highlights the central role of leadership commitment in shaping food safety culture, in line with prior research emphasising management-driven compliance dynamics [11].

The importance of leadership commitment is further illustrated by differences in how organizational roles perceive HACCP implementation challenges and emerging digital tools. Previous research has shown that personnel occupying different positions within HACCP-based food safety management systems may prioritize barriers differently, with frontline or food safety team members placing greater emphasis on production burdens, employee-related constraints, management attributes, and infrastructure, whereas leadership roles may prioritize different system-level concerns [47,48]. Indeed, food safety team leaders and team members have been shown to develop distinct perceptions of barriers even within the same food safety management system [47]. In the present study, this role-dependent pattern may also help explain the comparatively lower rating assigned by Production Staff to LLM-supported HACCP decision-making (Q21 = 3.50). Rather than indicating generalized scepticism toward AI, this finding may reflect differences in role-specific exposure, perceived usefulness, workflow compatibility, and the immediate operational relevance of AI-supported decision-making. This interpretation is consistent with evidence showing that food safety compliance is shaped by interacting organizational and human factors, including staff resistance, insufficient knowledge and training, managerial support, supervision, and resource limitations [48,49]. Similar role-dependent differences have also been observed in other standardized quality-management settings, where personnel closer to operational activities may perceive documentation and procedural requirements differently from senior management [50]. Importantly, factors such as workload, psychological safety, digital familiarity, organizational support, and trust in AI were not directly measured in the present study. They should therefore be regarded as plausible explanatory mechanisms rather than demonstrated mediators of the observed differences and warrant direct investigation in future studies [38,47,48,49,50].

Multiple studies indicate that documentation burden is consistently experienced more negatively by operational-level staff than by management, primarily due to its direct interference with core professional activities and time spent away from frontline tasks [51,52]. Frontline personnel frequently describe documentation either as an essential but time-consuming obligation or as a perceived administrative burden disconnected from practical risk prevention, whereas senior managers tend to emphasise its role in accountability, compliance, and quality assurance. Although HACCP-specific studies addressing documentation perceptions remain limited, record keeping is explicitly identified as a central component of HACCP systems, serving to demonstrate adherence and control verification [7,53].

Studies examining HACCP compliance consistently report that financial limitations, lack of training, and insufficient managerial support operate as interdependent barriers rather than competing ones [54,55,56]. Cost-related pressures may restrict investments in training, digital tools, and system optimization, while human resource limitations amplify the perceived workload associated with documentation and monitoring activities. This interdependence supports the interpretation that documentation intensity and financial constraints jointly shape practitioners’ experiences of HACCP implementation, particularly in resource-constrained hospitality settings.

Multiple studies highlight that cost-related pressures frequently result in partial or reactive implementation of food safety systems, rather than proactive risk prevention strategies [57,58]. These constraints tend to intensify during periods of economic stress, amplifying the trade-offs between regulatory compliance and operational viability.

The moderate but significant correlation between documentation burden and financial costs (r = 0.41, *p* < 0.05) suggests that administrative workload and economic constraints are experienced as mutually reinforcing pressures. These interdependencies appear to have been intensified during the COVID-19 pandemic, which increased operational costs and documentation demands through stricter hygiene and monitoring requirements [17,19]. At the same time, the strong alignment between legislative compliance and business reputation (r = 0.51, *p* < 0.01) indicates that regulatory adherence is framed by practitioners not merely as a legal obligation, but as a strategic investment in organizational credibility and trust [59,60,61,62].

The comparative analysis between human responses and the LLM-derived benchmark further extends these findings by revealing systematic patterns of convergence and divergence. While the one-sample t-test demonstrated close alignment between the ChatGPT 4.1 and human professionals regarding perceived HACCP benefits (*p* = 0.011), statistically significant deviations were observed in both implementation barriers (*p* < 0.001) and digital readiness (*p* < 0.001). In particular, the LLM baseline for AI readiness (4.39) exceeded the corresponding human mean score (4.12), suggesting a tendency toward higher assessments of the practical feasibility of digital HACCP adoption under the present experimental conditions. This divergence likely reflects limited sensitivity to contextual constraints such as technological lag, resource limitations, and continued reliance on manual record keeping practices prevalent in Southern European hospitality settings [18].

This technological lag limits real-time monitoring, increases the likelihood of documentation errors, and constrains the scalability of food safety management systems in complex operational environments. Importantly, statistical significance should not be interpreted as equivalent to practical significance. Although statistically significant Human–ChatGPT differences were identified, the observed absolute score differences were generally modest and should therefore be interpreted cautiously, as they indicate systematic perceptual divergence rather than necessarily substantial disagreement in everyday HACCP practice. In the context of developing and operating an HACCP system, modest overestimations of digital readiness or AI-driven mitigation of barriers could lead to operational overestimation if not filtered through human expertise and insight [63,64,65,66,67]. The practical value of this comparison lies primarily in identifying domains in which ChatGPT-generated assessments converge with, or systematically diverge from, professional perceptions, thereby informing the design of human-supervised AI tools for HACCP training, documentation support, and preliminary decision support. These findings should not be interpreted as evidence that LLMs can independently perform operational HACCP tasks. Validation through scenario-based hazard analysis, CCP identification, critical-limit setting, and corrective-action assessment is required before such applications can be considered.

Analysis of the ChatGPT 4.1-generated justifications indicates that model responses predominantly reflect standardized regulatory frameworks and generalized best-practice assumptions, whereas human participants consistently emphasise operational feasibility, contextual adaptation, and the necessity of human-in-the-loop oversight. This contrast highlights the complementary nature of human experiential knowledge and AI-generated reasoning, particularly in safety-critical decision-making contexts. Importantly, the results also point toward a pragmatic pathway for digital transition. The strong association between perceived AI usefulness for training and willingness to participate in AI-based programs (r = 0.68, *p* < 0.01) positions training-oriented AI applications as a credible and socially acceptable entry point for digital transformation within HACCP systems. The observed differences across roles suggest that perceptions of HACCP barriers and digital readiness vary between operational and regulatory contexts. The LLM appears to align more closely with regulatory/theoretical frameworks, which may partly explain divergences from operational, frontline professional responses [68,69,70,71,72].

In summary, several structural divergences emerge when viewed from a broader perspective. The discrepancies between human judgement and AI-generated reasoning underscore the inherent limitations of relying solely on algorithmic outputs. While training Large Language Models on global datasets, technical manuals, and regulatory frameworks provides extensive generalized knowledge, these models often fail to detect the local nuances and operational realities that shape workforce behaviour in specific sectors, such as hospitality. In the present study, this is clearly evidenced by the divergent perceptions regarding barriers to HACCP implementation.

A risk of algorithmic bias appears to be present, favouring a ‘checklist and compliance’ orientation rather than a proactive food safety culture. Consequently, professional judgement must remain the primary authority in governance and ethical decision-making, with AI serving in a subsidiary, supportive role. As AI tools become increasingly integrated into industrial safety systems, their role should be clearly defined: primarily assisting in training and documentation validation while the human factor remains central to operational control, ethical responsibility and the contextual interpretation of risks [73,74,75]. Overall, the effective integration of AI into HACCP frameworks requires continuous validation against empirically grounded human perspectives, strong governance mechanisms, and leadership commitment to ensure that digital tools enhance—rather than replace—professional judgement in food safety management systems [76,77,78,79].

From a practical perspective, the present findings support a clearly bounded, human-supervised role for ChatGPT-4.1 in HACCP-related applications. Appropriate use cases may include staff training, synthesis of regulatory or technical information, documentation support, and preliminary decision support. Conversely, the tested model should not replace qualified professionals in safety-critical functions such as site-specific hazard identification, CCP determination, establishment of critical limits, interpretation of monitoring deviations, or selection of corrective actions. Any LLM-generated recommendation should be independently verified against applicable regulatory requirements, validated HACCP plans, and facility-specific operational conditions before implementation. This is particularly important because inaccurate, incomplete, or contextually inappropriate recommendations could result in ineffective control measures and potentially compromise food safety. Accordingly, human oversight and clearly defined accountability should remain integral to any LLM-assisted HACCP workflow. Future research should move beyond perception-based benchmarking toward controlled, scenario-based validation using real or realistically simulated HACCP decision cases with predefined expert or regulatory reference standards.

## 5. Conclusions and Limitations

This exploratory study acknowledges certain limitations, primarily the relatively small sample size (n = 90), purposive sampling strategy, and geographic specificity of the human dataset. The geographical distribution of participants was concentrated primarily in Attica and Central Macedonia, limiting the external validity of the findings. Accordingly, the results should not be directly extrapolated to other countries, cultural or regulatory contexts, or to other food-sector environments such as food manufacturing, retail, and healthcare food service, where HACCP implementation conditions may differ substantially. The observed Human–LLM differences should therefore be interpreted as context-specific findings rather than as universally generalisable patterns. Future studies should validate these findings using larger and more geographically diverse samples across different food industry sectors and regulatory environments. Accordingly, the tendency of the tested model to assign higher ratings to several HACCP barriers should be interpreted as a context-specific comparative finding rather than evidence of systematic LLM overestimation across populations or food service environments. Importantly, the observed Human–ChatGPT alignment refers exclusively to similarity in perceived ratings across the examined HACCP dimensions and should not be interpreted as evidence of equivalent professional judgement, HACCP decision quality, hazard identification accuracy, or effectiveness in real-world food safety practice, none of which were directly evaluated in the present study.

Furthermore, as LLM technology evolves rapidly, these results represent a specific technological snapshot. Thus, issues of output stability, inherent bias, and reproducibility remain critical open questions. Given the tendency of the ChatGPT 4.1 to overestimate implementation barriers and digital readiness relative to human responses and the identified “training–turnover association” in the Greek hospitality sector, ChatGPT 4.1 should be viewed as supportive tool rather than an autonomous risk assessment system. While it may offer potential for training, regulatory information synthesis, and documentation support, it cannot replace professional judgement and requires rigorous human-in-the-loop oversight to ensure safety-critical reliability. The benchmark-based statistical comparison does not explicitly model uncertainty across individual LLM generations. Future studies could incorporate run-level LLM variability using bootstrap, permutation, or hierarchical modelling approaches.

An additional limitation is that the computational comparison was conducted exclusively with ChatGPT 4.1. Consequently, the observed Human–AI convergence and divergence patterns are specific to this model and its configuration at the time of inference and should not be generalized to other LLMs. Future research should conduct cross-model validation using multiple LLM architectures to determine whether the observed patterns are model-specific or reproducible across different systems.

A final limitation is the inherent ‘contextual gap’ between the datasets. The tested model is trained on vast global repositories that represent idealized standards, whereas the human sample reflects the specific socio-economic conditions of the Greek hospitality sector. This may explain discrepancies where the AI aligns with theoretical best practices while human professionals prioritize practical, context-dependent challenges. Future research should explore multi-regional samples to further address this geographic and demographic variability.

Based on the findings of this study, several actionable recommendations can be proposed for stakeholders. For industry practitioners, LLMs should be utilized as documentation aids rather than autonomous decision-makers, employing a ‘human-in-the-loop’ approach where AI-generated drafts are rigorously validated by experienced professionals. Regulatory bodies should consider developing frameworks that emphasise the necessity for AI-assisted safety plans to explicitly account for local operational constraints. For researchers, the focus should shift toward developing context-aware prompting techniques tailored to specific operational environments.

Future studies should systematically compare zero-shot, context-enriched, few-shot, and retrieval-augmented generation (RAG) approaches to determine whether grounding LLMs in validated local regulatory, organizational, and sector-specific knowledge improves alignment with professional HACCP assessments.

Such efforts will facilitate the development of transparent, scalable digital tools capable of complementing professional expertise. Ultimately, the transition toward digital HACCP systems must prioritize educational value and leadership commitment, leveraging AI as a strategic entry point to foster a resilient food safety culture in resource-constrained environments.

## Figures and Tables

**Table 1:** Mean Scores of ChatGPT 4.1-Generated Responses Across Five Professional Roles (N = 30 runs per role).

Question No	Item	Consultant	Production Staff	Manager/ Owner	Auditor	Researcher/ Academic	Average Score
SECTION B— BARRIERS							
Q5	Financial constraints/Equipment renewal costs	4.63	4.37	4.87	4.47	4.70	4.61
Q6	Lack of staff motivation and commitment	4.70	4.87	4.43	4.60	4.50	4.62
Q7	High staff turnover	4.80	4.77	4.70	4.80	4.77	4.77
Q8	Limited technical expertise of staff	4.53	4.67	4.33	4.70	4.43	4.53
Q9	Time required for documentation (record keeping)	3.90	4.83	3.50	4.07	3.23	3.91
Q10	Management support and commitment	4.23	4.43	4.53	4.50	4.73	4.48
Q11	Insufficient prerequisite programs (PRPs)	4.30	4.10	4.17	4.87	4.67	4.42
Q12	Inability to ensure continuous training	4.67	4.73	4.40	4.77	4.83	4.68
Q13	Lack of specialized technical consultants	4.13	3.87	3.97	4.23	4.33	4.11
SECTION C— BENEFITS							
Q14	Improves compliance with legislation	4.70	4.40	4.60	4.97	4.90	4.71
Q15	Reduces the likelihood of customer complaints	4.53	4.30	4.77	4.67	4.50	4.55
Q16	Enhances business reputation and credibility	4.87	4.53	4.90	4.87	4.83	4.80
Q17	Increases customer satisfaction and trust	4.73	4.47	4.77	4.63	4.60	4.64
Q18	Promotes food safety culture among employees	4.63	4.40	4.73	4.87	4.93	4.71
SECTION D— AI/DIGITALIZATION							
Q19	AI can contribute to staff HACCP training	4.40	3.97	4.10	4.57	4.77	4.36
Q20	AI systems can assist with automatic monitoring of CCPs and PRPs	4.70	4.27	4.63	4.83	4.80	4.65
Q21	Large Language Models (LLMs) can support HACCP decision-making	4.37	3.90	4.13	4.67	4.73	4.36
Q22	Willingness to participate in AI-utilizing HACCP training program	4.23	3.7	4.07	4.33	4.6	4.19
Average Score		4.50	4.37	4.42	4.63	4.60	4,5

**Table 2:** Demographic and professional characteristics of the participants (n = 90).

Category	Characteristic	Percentage (%)/ Frequency
Experience in HACCP	<1 year	8%
	1–3 years	15%
	4–7 years	22%
	8–10 years	18%
	>10 years	37%
Organization Type	Mass Catering/Catering Units	42%
	Hotel Units	35%
	Consulting/Auditing Firms	12%
	Public Authorities/Regulatory Bodies	11%
Training	Certified HACCP Training	82%
	No Official Certification	18%
Professional role	HACCP Consultant/Auditor	33
	Quality or Food Safety Manager	32
	Manager/Business Owner	10
	Researcher/Academic	7
	Production/Kitchen Staff	5
	Other food safety–related roles (e.g., regulatory authority staff, assistants, inspectors):	3
Geographical region of professional activity	Attica	38
	Central Macedonia	24
	Eastern Macedonia and Thrace	6
	Peloponnese	5
	Crete	4
	Ionian islands	4
	Other regions (Sterea Ellada, North Aegean, South Aegean, Thessaly, Western Greece, Epirus, Western Macedonia)	9

**Table 3:** Human participant survey results: Mean scores per HACCP dimension and professionals (n = 90).

Question No	Item	Consultant	Production Staff	Manager/ Owner	Auditor	Researcher/ Academic	Average Score
SECTION B— BARRIERS							
Q5	Financial constraints/Equipment renewal costs	4.15	3.85	4.3	4.1	3.95	4.07
Q6	Lack of staff motivation and commitment	4.4	4.2	4.15	4.5	4.35	4.32
Q7	High staff turnover	4.55	4.6	4.45	4.7	4.65	4.59
Q8	Limited technical expertise of staff	4.2	4.1	3.95	4.35	4.25	4.17
Q9	Time required for documentation (record keeping)	4.1	4.45	4.25	4.15	3.8	4.15
Q10	Management support and commitment	4.35	4.05	4.4	4.55	4.45	4.36
Q11	Insufficient prerequisite programs (PRPs)	3.9	3.75	3.85	4.2	4.1	3.96
Q12	Inability to ensure continuous training	4.45	4.3	4.2	4.6	4.55	4.42
Q13	Lack of specialized technical consultants	3.65	3.4	3.55	3.8	3.75	3.63
SECTION C— BENEFITS							
Q14	Improves compliance with legislation	4.85	4.65	4.75	4.9	4.95	4.82
Q15	Reduces the likelihood of customer complaints	4.6	4.5	4.7	4.75	4.65	4.64
Q16	Enhances business reputation and credibility	4.75	4.6	4.85	4.8	4.8	4.76
Q17	Increases customer satisfaction and trust	4.7	4.55	4.8	4.75	4.7	4.7
Q18	Promotes food safety culture among employees	4.5	4.35	4.45	4.7	4.8	4.56
SECTION D— AI/DIGITALIZATION							
Q19	AI can contribute to staff HACCP training	4.1	3.8	3.9	4.25	4.45	4.1
Q20	AI systems can assist with automatic monitoring of CCPs and PRPs	4.35	3.95	4.15	4.5	4.6	4.31
Q21	Large Language Models (LLMs) can support HACCP decision-making	3.85	3.5	3.7	4.1	4.3	3.89
Q22	Willingness to participate in AI-utilizing HACCP training program	4.2	3.9	4.05	4.3	4.5	4.19
Average Score		4.50	4.37	4.42	4.63	4.60	4.5

**Table 4:** Comparative Statistical Analysis between Human Responses and LLM (ChatGPT 4.1) Outputs.

Question No	Item Description	LLM Average	Human Average	Δ (LLM − Human)
SECTION B— BARRIERS				
Q5	Financial constraints/Equipment renewal costs	4.61	4.07	0.54
Q6	Lack of staff motivation and commitment	4.62	4.32	0.3
Q7	High staff turnover	4.77	4.59	0.18
Q8	Limited technical expertise of staff	4.53	4.17	0.36
Q9	Time required for documentation (record keeping)	3.91	4.15	−0.24
Q10	Management support and commitment	4.48	4.36	0.12
Q11	Insufficient prerequisite programs (PRPs)	4.42	3.96	0.46
Q12	Inability to ensure continuous training	4.68	4.42	0.26
Q13	Lack of specialized technical consultants	4.11	3.63	0.48
Average		4.45	4.18	
SECTION C— BENEFITS				
Q14	Improves compliance with legislation	4.71	4.82	−0.11
Q15	Reduces the likelihood of customer complaints	4.55	4.64	−0.09
Q16	Enhances business reputation and credibility	4.8	4.76	0.04
Q17	Increases customer satisfaction and trust	4.64	4.7	−0.06
Q18	Promotes food safety culture among employees	4.71	4.56	0.15
Average		4.62	4.69	
SECTION D— AI/DIGITALIZATION				
Q19	AI can contribute to staff HACCP training	4.36	4.1	0.26
Q20	AI systems can assist with automatic monitoring of CCPs and PRPs	4.65	4.31	0.34
Q21	Large Language Models (LLMs) can support HACCP decision-making	4.36	3.89	0.47
Q22	Willingness to participate in AI-utilizing HACCP training program	4.19	4.19	0
Average		4.39	4.12	0.19

## Data Availability

The original contributions presented in this study are included in the article/supplementary material. Further inquiries can be directed to the corresponding author.

## Supplementary Materials

The following supporting information can be downloaded at https://www.mdpi.com/article/10.3390/foods15162916/s1, File S1: HACCP Implementation Questionnaire Greek-English version.
