# Supplementary material for: Can ChatGPT Reflect Professional HACCP Judgments? A Comparative Study in Hospitality Food Safety
Source: Foods. 2026 Aug 20;15(16):2916. doi: 10.3390/foods15162916 (PMC13511892; doi:10.3390/foods15162916)
Supplement: Supplementary file 1 [file foods-15-02916-s001.zip › File S1. HACCP Implementation Questionnaire Greek-English version.pdf]

foods

MDPI

Supplementary Material

Can ChatGPT Reflect Professional HACCP Judgments? A Comparative Study in Hospitality Food Safety

Despoina Maria Konstantinidi [1], Elisavet Stavropoulou [1], Agathangelos Stavropoulos [1], Chrysoula (Chrysa) Voidarou [2], Christina Tsigalou [1], Vassiliki Pitiriga [3] and Christos Stefanis [1,*]

[1] Laboratory of Hygiene and Environmental Protection, Medical School, Democritus University of Thrace, 68100 Alexandroupolis, Greece; despoinamaria.n.konstantinidi@gmail.com (DMK); elisabeth.stavropoulou@gmail.com (E.S.); angelostavrop@gmail.com (A.S.); ctsigalo@med.duth.gr (C.T.)
[2] Department of Agriculture, School of Agriculture, University of Ioannina, 47100 Arta, Greece; xvoidarou@uoi.gr
[3] Department of Microbiology, Medical School, University of Athens, Athens, Greece; vpitiriga@med.uoa.gr
* Correspondence: chris.stefanis@gmail.com

1. Questionnaire (Greek)

This document presents the full questionnaire utilized in the study, "The HACCP System: Development and Implementation in Mass Catering and Hotel Units," which aims to investigate perceptions, experiences, and challenges related to the development and practical application of the Hazard Analysis and Critical Control Points (HACCP) system in the Greek food service and hospitality sectors. The survey was specifically designed for and distributed to professionals, researchers, academics, and consultants with direct experience in the development, implementation, inspection, or study of HACCP systems, either through their operational work or scientific projects. The goal was to collect responses that reflect real-world practices and evidence-based opinions.

The questionnaire is structured into five main sections, covering 28 questions:

Section A (Questions 1-6): Participant Profile and Demographics (Role, years of HACCP experience, organization type, region, and training).

Section B (Questions 7-12): HACCP Implementation and Operational Effectiveness (Internal audits, plan effectiveness, personnel compliance, management support, staff training, and use of digital tools).

Section C (Questions 13-18): Barriers and Challenges in HACCP Implementation (Cost, staff turnover, workload, training deficiency, allergen management, and technological infrastructure).

Section D (Questions 19-23): Benefits and Impact of HACCP (Food quality, reduction of customer complaints, reputation, customer trust, and food safety culture).

Section E (Questions 24-27): Digital Perspectives and Artificial Intelligence (The role of AI and LLMs in HACCP training, monitoring, and decision-making).

Participation was voluntary and anonymous, with no collection of personal data. The collected data supports the findings and discussions presented in the main article.

"Το σύστημα HACCP: Ανάπτυξη και Εφαρμογή σε Μονάδες Μαζικής Εστίασης και Ξενοδοχεία"

Αγαπητέ/ή συμμετέχοντα,

Σκοπός του παρόντος ερωτηματολογίου είναι να διερευνήσει τις αντιλήψεις, τις εμπειρίες και τις προκλήσεις που σχετίζονται με την ανάπτυξη και εφαρμογή του συστήματος HACCP σε μονάδες μαζικής εστίασης και ξενοδοχειακές επιχειρήσεις.

Η έρευνα απευθύνεται σε επαγγελματίες, ερευνητές, ακαδημαϊκούς ή συμβούλους που έχουν ασχοληθεί με την ανάπτυξη, εφαρμογή, επιθεώρηση ή μελέτη συστημάτων HACCP, είτε μέσα από την εργασία τους σε επιχειρήσεις είτε μέσα από ερευνητικά και επιστημονικά έργα. Η εμπειρία και η γνώση του αντικειμένου είναι σημαντική, ώστε οι απαντήσεις να αντανακλούν πραγματικές πρακτικές και τεκμηριωμένες απόψεις.

Η συμμετοχή είναι ανώνυμη και εθελοντική, δεν συλλέγονται προσωπικά δεδομένα και η συμπλήρωση διαρκεί περίπου 3-5 λεπτά. Με τη συμπλήρωση του ερωτηματολογίου δηλώνετε τη συναίνεσή σας για συμμετοχή στην έρευνα.

Στοιχεία επικοινωνίας: Δρ. Χρήστος Στεφανής - 6947153707, Δέσποινα Μαρία Κωνσταντινίδη - 6942857387

Ενότητα Α: Προφίλ Συμμετέχοντα - Δημογραφικές Ερωτήσεις

1. Ποια από τις παρακάτω δηλώσεις περιγράφει καλύτερα την εμπειρία σας με την εφαρμογή του HACCP;* (Επιλέξετε τη φράση που σας αντιπροσωπεύει περισσότερο) (Να επισημαίνεται μόνο μία έλλειψη.)

☐ **Υπάρχει πλήρως εφαρμοσμένο σύστημα HACCP στη μονάδα όπου εργάζομαι**

☐ **Στη μονάδα όπου εργάζομαι, το HACCP εφαρμόζεται μερικώς ή βρίσκεται υπό ανάπτυξη**

☐ **Δεν εργάζομαι σε μονάδα, αλλά έχω ερευνητική ή επαγγελματική εμπειρία με την ανάπτυξη/εφαρμογή HACCP**

☐ **Δεν έχω εμπειρία με το HACCP**

2. Ποιος είναι ο κύριος ρόλος σας σε σχέση με τα συστήματα HACCP;* (Να επισημαίνεται μόνο μία έλλειψη.)

☐ **Υπεύθυνος/η ποιοτικού ελέγχου ή ασφάλειας τροφίμων**

☐ **Μέλος προσωπικού παραγωγής ή κουζίνας**

☐ **Διευθυντικό στέλεχος ή ιδιοκτήτης επιχείρησης**

☐ **Σύμβουλος ή επιθεωρητής HACCP**

☐ **Ερευνητής/Ακαδημαϊκός**

☐ **Άλλο:**

3. Πόσα χρόνια εμπειρίας έχετε συνολικά στον τομέα των συστημάτων HACCP;* (Να επισημαίνεται μόνο μία έλλειψη.)

☐ **<1 έτος**

☐ 1-3 έτη

☐ 4-7 έτη

☐ 8-10 έτη

☐ >10 έτη

4. Σε ποιο είδος οργανισμού/περιβάλλοντος σχετίζεται κυρίως η εμπειρία σας με το HACCP; (Να επισημαίνεται μόνο μία έλλειψη.)

☐ Ξενοδοχειακή μονάδα

☐ Μονάδα μαζικής εστίασης/ Catering

☐ Εργαστήριο έρευνας ή πανεπιστήμιο

☐ Συμβουλευτική/ φορέας πιστοποίησης

☐ Δημόσιος φορέας/ αρχή ελέγχου

☐ Άλλο:

5. Έχετε παρακολουθήσει επίσημη εκπαίδευση ή πιστοποίηση HACCP;* (Να επισημαίνεται μόνο μία έλλειψη.)

☐ Ναι

☐ Όχι

6. Σε ποια περιφέρεια δραστηριοποιείστε κυρίως; (Να επισημαίνεται μόνο μία έλλειψη.)

☐ Αττικής

☐ Κεντρική Μακεδονίας

☐ Θεσσαλίας

☐ Δυτικής Ελλάδας

☐ Κρήτης

☐ Ανατολικής Μακεδονίας και Θράκης

☐ Πελοποννήσου

☐ Στερεάς Ελλάδας

☐ Νοτίου Αιγαίου

☐ Ηπείρου

☐ Δυτικής Μακεδονίας

☐ Ιονίων Νήσων

☐ Βορείου Αιγαίου

Ενότητα Β - Εφαρμογή HACCP

(Κλίμακα: 1=Καθόλου έως 5=Πάρα πολύ. Οι ερωτήσεις αφορούν την εφαρμογή HACCP σε μονάδες εργασίας, με βάση την επαγγελματική ή ερευνητική εμπειρία των συμμετεχόντων)

7. Πόσο συχνά, σύμφωνα με την εμπειρία σας, διενεργούνται εσωτερικές επιθεωρήσεις ή έλεγχοι HACCP στις επιχειρήσεις με τις οποίες εργάζεστε ή συνεργάζεστε;* (Να επισημαίνεται μόνο μία έλλειψη.)

☐ 1 ☐ 2 ☐ 3 ☐ 4 ☐ 5

8. Πόσο αποτελεσματικό θεωρείτε το σχέδιο HACCP ως προς την προληπτική του ικανότητα να αποτρέψει την εμφάνιση κινδύνων (βιολογικών, χημικών, φυσικών) στα Σημεία Κρίσιμου Ελέγχου, προστατεύοντας τη δημόσια υγεία.* (Να επισημαίνεται μόνο μία έλλειψη.)

☐ 1 ☐ 2 ☐ 3 ☐ 4 ☐ 5

9. Πόσο εύκολη, σύμφωνα με την εμπειρία σας, θεωρείτε τη συμμόρφωση του προσωπικού στις διαδικασίες HACCP;* (Να επισημαίνεται μόνο μία έλλειψη.)

☐ 1 ☐ 2 ☐ 3 ☐ 4 ☐ 5

10. Σε ποιο βαθμό, σύμφωνα με την εμπειρία σας, η διοίκηση των επιχειρήσεων στηρίζει ενεργά την εφαρμογή του HACCP;* (Να επισημαίνεται μόνο μία έλλειψη.)

☐ 1 ☐ 2 ☐ 3 ☐ 4 ☐ 5

11. Πόσο επαρκής θεωρείτε, από την εμπειρία ή την έρευνά σας, την εκπαίδευση του προσωπικού στις αρχές HACCP;* (Να επισημαίνεται μόνο μία έλλειψη.)

☐ 1 ☐ 2 ☐ 3 ☐ 4 ☐ 5

12. Σε ποιο βαθμό, σύμφωνα με την επαγγελματική ή ερευνητική σας εμπειρία, χρησιμοποιούνται ψηφιακά εργαλεία για την παρακολούθηση του HACCP;* (Να επισημαίνεται μόνο μία έλλειψη.)

☐ 1 ☐ 2 ☐ 3 ☐ 4 ☐ 5

Ενότητα Γ - Εμπόδια και Προκλήσεις

(Κλίμακα: 1=Καθόλου σημαντικό έως 5=Πάρα πολύ σημαντικό. Αξιολογήστε τη σημασία των παρακάτω παραγόντων ως εμποδίων στην εφαρμογή του HACCP με βάση την επαγγελματική ή ερευνητική σας εμπειρία.)

13. Κόστος σχεδιασμού και εφαρμογής HACCP* (Να επισημαίνεται μόνο μία έλλειψη.)

☐ 1 ☐ 2 ☐ 3 ☐ 4 ☐ 5

14. Έλλειψη ή συχνή αλλαγή προσωπικού* (Να επισημαίνεται μόνο μία έλλειψη.)

☐ 1 ☐ 2 ☐ 3 ☐ 4 ☐ 5

15. Έλλειψη χρόνου λόγω αυξημένου φόρτου εργασίας* (Να επισημαίνεται μόνο μία έλλειψη.)

☐ 1 ☐ 2 ☐ 3 ☐ 4 ☐ 5

16. Ελλιπής εκπαίδευση και ευαισθητοποίηση του προσωπικού* (Να επισημαίνεται μόνο μία έλλειψη.)

☐ 1 ☐ 2 ☐ 3 ☐ 4 ☐ 5

17. Δυσκολία στη διαχείριση αλλεργιογόνων ή ειδικών διατροφικών αναγκών* (Να επισημαίνεται μόνο μία έλλειψη.)

☐ 1 ☐ 2 ☐ 3 ☐ 4 ☐ 5

18. Έλλειψη ψηφιακών ή τεχνολογικών υποδομών* (Να επισημαίνεται μόνο μία έλλειψη.)

☐ 1 ☐ 2 ☐ 3 ☐ 4 ☐ 5

Ενότητα Δ - Οφέλη και Αντίκτυπος

(Κλίμακα: 1=Διαφωνώ απόλυτα έως 5=Συμφωνώ απόλυτα. Αξιολογήστε τον βαθμό στον οποίο το HACCP συμβάλλει στα παρακάτω, με βάση την εμπειρία ή την παρατήρησή σας.)

19. Το HACCP συμβάλλει στη βελτίωση της ποιότητας των τροφίμων* (Να επισημαίνεται μόνο μία έλλειψη.)

☐ 1 ☐ 2 ☐ 3 ☐ 4 ☐ 5

20. Το HACCP μειώνει την πιθανότητα παραπόνων πελατών* (Να επισημαίνεται μόνο μία έλλειψη.)

☐ 1 ☐ 2 ☐ 3 ☐ 4 ☐ 5

21. Το HACCP ενισχύει τη φήμη και την αξιοπιστία της επιχείρησης* (Να επισημαίνεται μόνο μία έλλειψη.)

☐ 1 ☐ 2 ☐ 3 ☐ 4 ☐ 5

22. Το HACCP αυξάνει την ικανοποίηση και την εμπιστοσύνη των πελατών* (Να επισημαίνεται μόνο μία έλλειψη.)

☐ 1 ☐ 2 ☐ 3 ☐ 4 ☐ 5

23. Το HACCP προάγει την κουλτούρα ασφαλείας τροφίμων μεταξύ των εργαζόμενων* (Να επισημαίνεται μόνο μία έλλειψη.)

☐ 1 ☐ 2 ☐ 3 ☐ 4 ☐ 5

Ενότητα Ε - Ψηφιακές προοπτικές και Τεχνητή Νοημοσύνη

(Κλίμακα: 1=Διαφωνώ απόλυτα έως 5=Συμφωνώ απόλυτα.)

24. Η τεχνητή νοημοσύνη (AI) μπορεί να συμβάλει στην εκπαίδευση του προσωπικού για το HACCP* (Να επισημαίνεται μόνο μία έλλειψη.)

☐ 1 ☐ 2 ☐ 3 ☐ 4 ☐ 5

25. Τα συστήματα AI μπορούν να βοηθήσουν στην αυτόματη παρακολούθηση κρίσιμων σημείων ελέγχου (CCPs) και προαπαιτούμενα* (Να επισημαίνεται μόνο μία έλλειψη.)

☐ **1** ☐ **2** ☐ **3** ☐ **4** ☐ **5**

26. Η χρήση εργαλείων όπως τα Large Language Models (LLMs) μπορεί να υποστηρίξει τη λήψη αποφάσεων στο HACCP* (Να επισημαίνεται μόνο μία έλλειψη.)

☐ **1** ☐ **2** ☐ **3** ☐ **4** ☐ **5**

27. Θα επιθυμούσα να συμμετάσχω σε πρόγραμμα εκπαίδευσης HACCP που αξιοποιεί Τεχνητή Νοημοσύνη* (Να επισημαίνεται μόνο μία έλλειψη.)

☐ **1** ☐ **2** ☐ **3** ☐ **4** ☐ **5**

28. Ποιες θεωρείτε τις σημαντικότερες προτάσεις βελτίωσης για την αποτελεσματική εφαρμογή του HACCP στη μονάδα σας;

Ανοιχτή Ερώτηση (Προαιρετική) - (Πλαίσιο απάντησης)

The HACCP System: Development and Implementation in Mass Catering and Hotel Units

Dear Participant,

The purpose of this questionnaire is to investigate perceptions, experiences, and challenges related to the development and implementation of the Hazard Analysis and Critical Control Points (HACCP) system in mass catering and hotel establishments.

This survey is addressed to professionals, researchers, academics, and consultants who have been involved in the development, implementation, auditing, or study of HACCP systems, either through professional practice or scientific projects. Relevant experience is important to ensure that responses reflect real-world practices and evidence-based perspectives.

Participation in this study is voluntary and anonymous. No directly identifiable personal data are collected, and completion of the questionnaire requires approximately 3–5 minutes.

By completing and submitting the questionnaire, you confirm that you have read the above information and provide your informed consent to participate in the study and for the anonymized results to be used for scientific research and potential publication.

Contact information:
Dr. Christos Stefanis
Despoina Maria Konstantinidi

Section A – Participant Profile (Demographic Questions)

Which of the following statements best describes your experience with HACCP implementation? (Select one)

☐ **A fully implemented HACCP system operates in the unit where I work**
☐ **HACCP is partially implemented or currently under development in my unit**

☐ I do not work in an operational unit, but I have research or professional experience with HACCP development/implementation
☐ I have no experience with HACCP

What is your primary professional role in relation to HACCP systems? (Select one)

☐ HACCP Consultant
☐ Production or Kitchen Staff Member
☐ Manager or Business Owner
☐ HACCP Auditor
☐ Researcher / Academic
☐ Other: _______

How many total years of experience do you have in HACCP systems? (Select one)

☐ <1 year
☐ 1–3 years
☐ 4–7 years
☐ 8–10 years
☐ >10 years

In which type of organization is your HACCP-related experience primarily based? (Select one)

☐ Hotel unit
☐ Mass catering / Catering unit
☐ Research laboratory or university
☐ Consulting firm / Certification body
☐ Public authority / Regulatory body
☐ Other: _______

Have you attended official HACCP training or certification? (Select one)

☐ Yes
☐ No

In which geographical region do you primarily operate? (Select one)

☐ Attica
☐ Central Macedonia
☐ Thessaly
☐ Western Greece
☐ Crete
☐ Eastern Macedonia and Thrace
☐ Peloponnese
☐ Central Greece
☐ South Aegean
☐ Epirus
☐ Western Macedonia
☐ Ionian Islands
☐ North Aegean

Section B – Barriers to HACCP Implementation

Scale: 1 = Not important | 5 = Very important

Q5. Financial constraints / Cost of HACCP design and implementation
☐ 1 ☐ 2 ☐ 3 ☐ 4 ☐ 5

Q6. Lack of staff motivation and commitment
☐ 1 ☐ 2 ☐ 3 ☐ 4 ☐ 5

Q7. High staff turnover
☐ 1 ☐ 2 ☐ 3 ☐ 4 ☐ 5

Q8. Limited technical expertise of staff
☐ 1 ☐ 2 ☐ 3 ☐ 4 ☐ 5

Q9. Time required for documentation and record-keeping
☐ 1 ☐ 2 ☐ 3 ☐ 4 ☐ 5

Q10. Management support and commitment
☐ 1 ☐ 2 ☐ 3 ☐ 4 ☐ 5

Q11. Insufficient prerequisite programs (PRPs)
☐ 1 ☐ 2 ☐ 3 ☐ 4 ☐ 5

Q12. Inability to ensure continuous training
☐ 1 ☐ 2 ☐ 3 ☐ 4 ☐ 5

Q13. Lack of specialized technical consultants
☐ 1 ☐ 2 ☐ 3 ☐ 4 ☐ 5

Section C – Benefits and Impact

Scale: 1 = Strongly disagree | 5 = Strongly agree

14. HACCP contributes to improving compliance with legislation
☐ 1 ☐ 2 ☐ 3 ☐ 4 ☐ 5

15. HACCP reduces the likelihood of customer complaints
☐ 1 ☐ 2 ☐ 3 ☐ 4 ☐ 5

16. HACCP enhances business reputation and credibility
☐ 1 ☐ 2 ☐ 3 ☐ 4 ☐ 5

17. HACCP increases customer satisfaction and trust
☐ 1 ☐ 2 ☐ 3 ☐ 4 ☐ 5

18. HACCP promotes a food safety culture among employees
☐ 1 ☐ 2 ☐ 3 ☐ 4 ☐ 5

Section D – Digital Readiness and Artificial Intelligence

Scale: 1 = Strongly disagree | 5 = Strongly agree

Q19. Artificial Intelligence (AI) can contribute to staff HACCP training
☐ 1 ☐ 2 ☐ 3 ☐ 4 ☐ 5

Q20. AI systems can assist in automatic monitoring of CCPs and PRPs
☐ 1 ☐ 2 ☐ 3 ☐ 4 ☐ 5

Q21. Large Language Models (LLMs) can support HACCP decision-making
☐ 1 ☐ 2 ☐ 3 ☐ 4 ☐ 5

Q22. Willingness to participate in AI-supported HACCP training
☐ 1 ☐ 2 ☐ 3 ☐ 4 ☐ 5

2. Complete Persona-Based Prompting Protocol

2.1. Persona/Role Instructions

Persona	Professional Role	Persona/Role Instruction (English Translation)
**Persona 1**	Greek Food Safety Consultant	"Answer as a Greek Food Safety Consultant with over 6 years of experience in hotels and catering units in Greece, possessing full knowledge of EU and Greek legislation, actual operational challenges, and common HACCP compliance issues."
**Persona 2**	Production/Kitchen Staff (Cook/Chef)	"Answer as a production/kitchen staff member with over 6 years of experience in Greek catering and hospitality, knowledgeable about daily practical implementation challenges of HACCP, legislative requirements, and operational hurdles in production."
**Persona 3**	Manager/Business Owner	"Answer as an executive manager or business owner with over 6 years of experience in hospitality and catering in Greece, with direct responsibility for HACCP, resource allocation, staffing, and regulatory compliance."
**Persona 4**	HACCP Inspector/Auditor	"Answer as a HACCP inspector or auditor with over 6 years of experience auditing hotels/restaurants in Greece, enforcing strict compliance with HACCP requirements, with experience in common violations and corrective actions."
**Persona 5**	Researcher/Academic	"Answer as a researcher/academic with over 6 years of research experience in HACCP, food safety science, and hospitality operations, applying an objective and analytical approach based on literature and empirical data."

3. LLM Configuration and Access Conditions

Model: GPT-4.1
Interface: ChatGPT web interface (OpenAI, San Francisco, CA, USA)
Inference period: May 2026
API: Not used
API version/settings: Not applicable
API-specific model snapshot: Not applicable

System instructions:. No researcher-defined system prompt was used. All researcher-controlled persona, context, task, response-scale, and output-format instructions are reproduced in full in Sections 2-4.
Model designation: GPT-4.1, as displayed to the researchers through the interface at the time of inference.

4. Execution Template Applied to Each Questionnaire Item

Role Description:
[Persona-specific role instruction reported in Section 2.1]

Context (Original Greek Input):
«Σου δίνω μια δήλωση από ερωτηματολόγιο που αξιολογεί την εφαρμογή του HACCP σε ξενοδοχεία και μονάδες μαζικής εστίασης στην Ελλάδα και τη Νότια Ευρώπη. Δώσε μια απάντηση κλίμακας Likert από 1 έως 5, εκφράζοντας την τεκμηριωμένη επαγγελματική σου κρίση σύμφωνα με τον επαγγελματικό ρόλο που καθορίστηκε παραπάνω.»

Response Scale (Original Greek Input):
«1 = Καθόλου / Διαφωνώ απόλυτα, 5 = Πάρα πολύ / Συμφωνώ απόλυτα.»

Output Instructions (Original Greek Input):
«Απάντησε αυστηρά χρησιμοποιώντας δύο γραμμές:

1. Βαθμολογία (1–5): X
2. Αιτιολόγηση: [σύντομη αιτιολόγηση 1–2 προτάσεων].»

Item Statement:
[Κείμενο της εκάστοτε ερώτησης του ερωτηματολογίου]

English Translation

Context: "You are given a statement from a questionnaire assessing HACCP implementation in hotels and mass-catering establishments in Greece and Southern Europe. Provide a Likert-scale response from 1 to 5, expressing your evidence-informed professional judgement according to the professional role defined above."

Response Scale: "1 = Not at all / Strongly disagree; 5 = Very much / Strongly agree."

Output Instructions: "Respond strictly using two lines:

1. Score (1–5): X
2. Justification: [brief justification of 1–2 sentences]."

Item Statement:
[English text of the corresponding questionnaire item]

Disclaimer/Publisher's Note: The statements, opinions and data contained in all publications are solely those of the individual author(s) and contributor(s) and not of MDPI and/or the editor(s). MDPI and/or the editor(s) disclaim responsibility for any injury to people or property resulting from any ideas, methods, instructions or products referred to in the content.
